# Heterogeneous Persister Cells Formation in *Acinetobacter baumannii*

**DOI:** 10.1371/journal.pone.0084361

**Published:** 2013-12-31

**Authors:** Valdir Cristóvão Barth, Belisa Ávila Rodrigues, Grasiela Daiane Bonatto, Stephanie Wagner Gallo, Vany Elisa Pagnussatti, Carlos Alexandre Sanchez Ferreira, Sílvia Dias de Oliveira

**Affiliations:** 1 Laboratório de Imunologia e Microbiologia, Faculdade de Biociências, Pontifícia Universidade do Rio Grande do Sul, Porto Alegre, RS, Brasil; 2 Departamento de Microbiologia do Laboratório de Patologia Clínica, Hospital São Lucas, Pontifícia Universidade do Rio Grande do Sul, Porto Alegre, RS, Brasil; University of California Merced, United States of America

## Abstract

Bacterial persistence is a feature that allows susceptible bacteria to survive extreme concentrations of antibiotics and it has been verified in a number of species, such as *Escherichia coli*, *Pseudomonas aeruginosa*, *Staphylococcus* spp., *Mycobacterium* spp. However, even though *Acinetobacter baumannii* is an important nosocomial pathogen, data regarding its persistence phenotype are still lacking. Therefore, the aim of this study was to evaluate the persistence phenotype in *A*. *baumannii* strains, as well as its variation among strains after treatment with polymyxin B and tobramycin. Stationary cultures of 37 polymyxin B-susceptible clinical strains of *A*. *baumannii* were analyzed for surviving cells after exposure to 15 µg/mL of polymyxin B for 6 h, by serial dilutions and colony counting. Among these, the 30 tobramycin-susceptible isolates also underwent tobramycin treatment at a concentration of 160 µg/mL and persister cells occurrence was evaluated equally. A high heterogeneity of persister cells formation patterns among isolates was observed. Polymyxin B-treated cultures presented persister cells corresponding from 0.0007% to 10.1% of the initial population and two isolates failed to produce detectable persister cells under this condition. A high variability could also be observed when cells were treated with tobramycin: the persister fraction corresponded to 0.0003%–11.84% of the pre-treatment population. Moreover, no correlation was found between persister subpopulations comparing both antibiotics among isolates, indicating that different mechanisms underlie the internal control of this phenotype. This is the first report of persister cells occurrence in *A*. *baumannii*. Our data suggest that distinct factors regulate the tolerance for unrelated antibiotics in this species, contrasting the multi-drug tolerance observed in other species (eg. dormancy-mediated tolerance). Supporting this observation, polymyxin B – an antibiotic that is believed to act on non-dividing cells as well – failed to eradicate persister cells in the majority of the isolates, possibly reflecting a disconnection between persistence and dormancy.

## Introduction

*Acinetobacter baumannii* is a nosocomial pathogen responsible for numerous deaths. Although much has been elucidated about its resistance phenotype and mechanisms, very little is known about the persistence phenotype in this species. Persistence is a feature already observed in some important species, such as *Escherichia coli* [1], *Pseudomonas aeruginosa* [2], *Staphylococcus aureus* [3] and *Mycobacterium* spp. [4]. Indeed, these studies show that strains phenotypically susceptible to antibiotics are not effectively eliminated upon exposure to high doses of those drugs. The remaining subpopulations after this treatment are called persister cells [5], in order to differentiate them from resistant cells [3], and are believed to be responsible for the recalcitrant nature and therapy unresponsiveness of several chronic infections [6]. Unlike resistant strains, in such cells there is not a specific genetic resistance mechanism, being initially inferred as a general regulation in bacterial metabolism leading to a dormancy state, which possibly reduces the antibiotic action through the diminished activity or availability of its target [5], [7], [8]. However, recent studies suggest that the persistence phenotype is not directly linked to dormancy [9], [10]. The molecular determinants for the formation of persister cells are still not clear and seem to involve different mechanisms implicated in stress responses, such as the stringent response to starvation and regulation of oxidative stress [11]–[13]. Additionally, mathematical models suggest that an interaction of multiple toxin-antitoxin (TA) systems together may regulate the presence and intensity of this tolerance phenotype [14]. Supporting this idea, some TA systems have been found to impact significantly in the composition and quantity of the antibiotic-tolerant subpopulation of a susceptible strain [7], [13], [15]. Most studies on that matter, however, have been performed using *E*. *coli* strains and not much is described for other clinically important species. To our knowledge, the ability to form persister cells has not yet been reported in *A*. *baumannii*, even though it is a major nosocomial pathogen that is often unresponsive to treatment. Therefore, the aim of this study was to verify the persistence phenotype in *A*. *baumannii* strains, as well as the variation of this phenotype among strains following antimicrobial exposure.

## Materials and Methods

### Bacterial Strains

Thirty-seven strains of *A*. *baumannii* were isolated as part of routine diagnostic testing and donated by the Microbiology Department of São Lucas Hospital, Porto Alegre, RS, Brazil, during January to September 2012. No information was retrieved along with the bacterial strains and the anonymity of the samples was respected throughout the study. The susceptibility to tobramycin and polymyxin B were previously evaluated by disk diffusion method and microdilution, respectively, according to the Clinical and Laboratory Standards Institute’s (CLSI) recommendations [16]. All 37 strains presented susceptibility to polymyxin B, 30 of which were also susceptible to tobramycin. The susceptibility to tobramycin was confirmed by the assessment of the Minimum Inhibitory Concentration (MIC) (Table S1). All strains evaluated in the current study presented unique profiles when analyzed by Pulsed-Field Gel Electrophoresis, antimicrobial susceptibility phenotype, persistence formation level, and presence of *bla*_VIM_, *bla*_OXA-23_ and *int*I2 (data not shown).

### Formation of Persister Cells

Persister cells from susceptible strains were estimated under exposure to polymyxin B (n = 37) and tobramycin (n = 30). All strains were cultivated for approximately 18 h on LB broth and initial cell concentration was measured by serial decimal dilutions proceeded by plating three 10-µL drops of each dilution on nutrient agar surface. In order to assess a death curve, polymyxin B or tobramycin was added to the media to a final concentration of 15 µg/mL (approximately 5× the highest MIC found) or 160 µg/mL (10× the MIC for a strain to be considered resistant, according to CLSI [17]). An aliquot was removed every 1.5 h, for 6 h, and washed using sterile 0.85% saline to remove the drug. The number of Colony Forming Units per milliliter (CFU/mL) of the surviving fraction was estimated by decimal serial dilutions and drop plating.

The final fraction of persisters for both unrelated antibiotics was estimated considering the pre-treatment cell counts and the surviving cells after the 6 h incubation. All experiments were performed in triplicate. Persister cells were re-cultivated into a sterile LB with the same concentration of antibiotic in order to discard the possibility of resistant mutant selection.

### Statistical Analysis

For frequency distribution charts, the persister producer strains were grouped in 6 classes (number defined by the rounded square root of the sample size).

The association between the persister cell formation upon exposure to both drugs as well as between persister formation and the MIC value for each strain were analyzed using Spearman’s rho (*r_s_*) test. All analyses were performed using SPSS® Statistics version 22 (IBM), using the significance level of 0.05.

## Results and Discussion

All clinical strains tested were susceptible to polymyxin B, presenting MIC values ranging from 0.5 to 2 µg/mL. Thirty-five (94.6%) of the isolates showed detectable persister cells formation, yet in varying intensities, upon polymyxin B exposure, displaying classic biphasic death curves (data not shown). Such high percentage of persister cells formation positive strains indicates that such phenotype may be widely distributed among *A*. *baumannii* strains, especially in the hospital settings. Surprisingly, we have found two isolates that failed to produce detectable levels of persister cells under these experimental conditions. Considering that these samples presented very low MIC values (0.5 µg/mL), the lack of persister cells could also be due to remaining inhibitory levels of antibiotic within the first dilutions, which prevented regrowth and, consequently, quantification of the few remaining persister cells on the agar plate. However, even after multiple saline washes associated to lower initial levels of polymyxin B (2.5 µg/mL instead of 15 µg/mL), they were still not able to produce detectable quantities of persister cells. The absence of persister cells in wild populations appears to be unusual and unreported in the literature so far, since the two studies that also have used a quantitative approach with more than one strain described all isolates as persister producers [1], [18]. This finding reveals that such putative persister genotype may be absent or have its expression silenced in some strains, not being crucial for bacterial viability.

**Figure 1:**
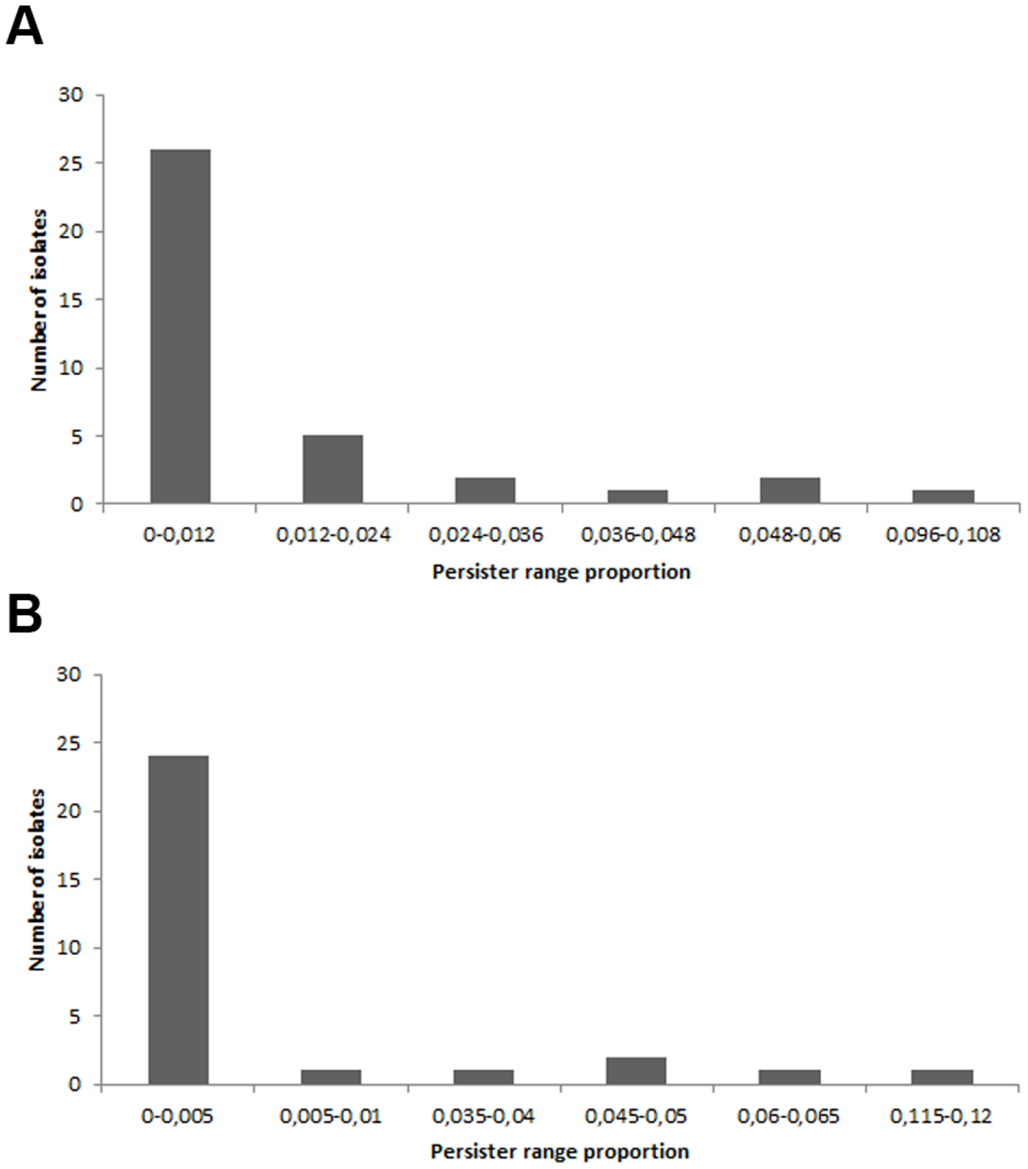
Frequency distribution of the persister fractions. Persister producer strains were grouped in 6 classes (defined by the square root of sample size) according to their persister fraction in the population after polymyxin B (a) or tobramycin (b) exposure. Most isolates presented persister fractions that corresponded to less than 1.2% and 0.5% of the total population for polymyxin B and tobramycin, respectively.

Concerning to the magnitude of persister cell formation, it was possible to notice a very heterogeneous pattern among the polymyxin B-tolerant persisters, since the size of these subpopulations fluctuated greatly from 0.0007% to 10.1% of the original pretreated population, which showed to be uncorrelated to the MIC values (*r_s_* = 0.081, *p* = 0.63). Indeed, 70.2% presented a persister cells fraction corresponding to up to 1.2% of the initial population (Figure 1a). This diversity in the phenotype intensity may indicate differential expression levels or number of non-redundant genetic determinants or distinct genes with greater potency in stimulating persistence in those isolates that showed higher persister cells formation. Such determinants are still being revealed in other species, such as *E*. *coli*, which appears to have a combination of TA modules as the strongest candidates to modulate this type of antibiotic tolerance [13], [14]. However, the genetic background participating in the development of this feature is yet to be determined in *A*. *baumannii*. Recently, Jurenaite et al. [19] have demonstrated computationally the presence of several type II TA modules integrated into *A*. *baumannii* chromosome and plasmids, including the HipAB, the module most associated with persistence in *E*. *coli*. Nonetheless, the authors have not established its role in persistence nor demonstrated its full functionality.

**Figure 2:**
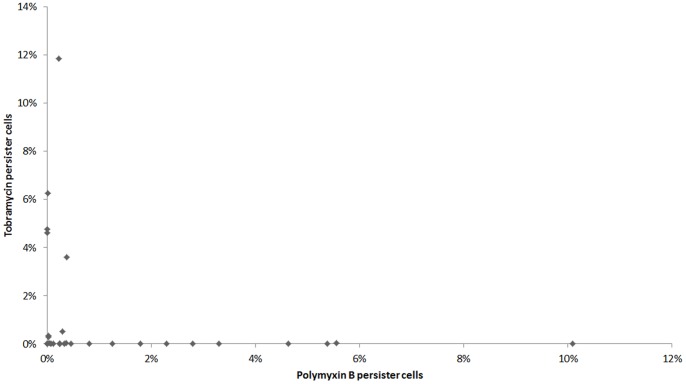
Correlation between polymyxin B and tobramycin persister cells in the sample population.

Although we found a high prevalence of persistence among the isolates, it must be considered that they have a nosocomial origin, therefore from an environment with constant selective pressure imposed by antibiotic therapy. Therefore, it is possible that such feature and its genetic determinants might have been selected by the antimicrobial drug pressure. Such phenotype perpetuation could also arise from selective pressures other than polymyxin B use, via cross effects with other antimicrobials or chemicals, as the presence of a generalized tolerance to multiple antimicrobials in some persistence reports has been demonstrated [7], [20]. Thus, we selected the 30 tobramycin susceptible strains among the 37 isolates tested for persister formation with polymyxin B, in order to evaluate such assumption to *A*. *baumannii* as well. Tobramycin-mediated persistence could be detected in 27 samples, including one that had not formed any detectable persister cells in the polymyxin B assay. Among the three isolates that failed to form persisters upon exposure to tobramycin, one had not shown any persistence level in the polymyxin B assay either. The persister subpopulation to tobramycin varied greatly from 0.0003% to 11.84% of total population, also forming a biphasic death curve (data not shown), and no correlation was found between MIC values and persister cells formation among the isolates for tobramycin either (*r_s_* = 0.3, *p* = 0.1). Although persistence could be observed after selection with both antibiotics tested for most isolates, there was no significant correlation (*r_s_* = −0.182, *p* = 0.336) between the intensity of persistence formation to polymyxin B and tobramycin (Figure 2). It must also be noted that the proportion of persister cells formation described here for both drugs are similar to those reported under similar exposure time in other species and antibiotics, for example, *E*. *coli* upon exposure to ampicillin [18] and *Listeria monocytogenes* using norfloxacin [21]. Drug-related variations in the proportion of persister cells formation among strains might also point to the need to clarify whether there is indeed an association of genetic determinants for persistence (eg. specific sets of TA modules) or their expression levels when cells are exposed to different drugs. Still, further analyses are needed to evaluate such assumption. The premise that dormancy justifies the persistence phenotype derives largely from experimentations in which single strains (commonly *E*. *coli* K-12-derived strains) presented similar persister levels upon exposure to distinct antibiotics [9], [13]. However, similar variations to those found in this study were also observed in strains of *E*. *coli* retrieved from environmental samples, evidencing a possible strain-to-strain discrepancy [18]. Our findings corroborate the data presented by Hofsteenge et al. [18] that also revealed an unexpected lack of correlation of persister cells formation between antimicrobials, even those with very similar mechanisms of action (ie. ciprofloxacin and nalidixic acid), substantiating the theory that dormancy alone is not sufficient to fully explain a state of multi-drug tolerance. In fact, it has been recently shown that even though most persister cells lie on a non-growing fraction, actively dividing cells are also part of the persister subpopulation and that only a small portion of dormant cells are actual persisters [9].

Furthermore, to our knowledge, this is the first report using polymyxin B to evaluate persister cells. Polymyxins interact initially with lipid components of the cell wall (LPS) and cell membrane, targets that theoretically are exposed and susceptible to the antimicrobial action even in cases of non-growing states, and this interaction constitutes a necessary step for bacterial killing [22]. Therefore, unlike most other antibiotics, polymyxin B should effectively kill cells despite their cell cycle stage (non-dividing or actively growing). The fact that we observed high persistence even after polymyxin B treatment also implies that other determinants that are independent of those from tobramycin-selected persisters are involved, perhaps specific for this drug, acting on other steps of polymyxin B killing. Recent reports suggest that polymyxin B (as well as many other bactericidal antimicrobials, including aminoglycosides, eg. tobramycin, which blocks protein synthesis) kill cells through the production of reactive oxygen species by a still unclear manner instead of directly blocking their targets [23], [24]. If this is the case, controlling ROS production, and not dormancy itself, might explain at least in part the tolerance to polymyxins. Nonetheless, data from recent analyses show certain controversy and are under debate [25]–[27], which reinforces the need to clarify the actual implication of these results.

## Conclusion

The high prevalence of *A. baumannii* persister cells producers in nosocomial strains following polymyxin B exposure points to a new degree of concern upon infection and reinfection on hospital environments. Also, qualitative and quantitative data regarding the diverseness of this phenotype and correlation between two mechanistically unrelated antimicrobial agents in this species showed here cannot be thoroughly explained by the information currently available in the scientific literature. The lack of data regarding TA modules in *A*. *baumannii*, for instance, limits the full understanding of the molecular background possibly related to persistence induction in this species, requiring a further emphasis in this research field. Additionally, the comprehension of the basis of persistence may contribute to the development of alternative strategies aiming the eradication of persister cells in chronic infections.

## Supporting Information

Table S1 **Minimum Inhibitory Concentration to polymyxin B and tobramycin in the isolates used in this study.**
(DOCX)
