# Supplementary material for: Heterogeneous Persister Cells Formation in Acinetobacter baumannii
Source: PLoS One. 2013 Dec 31;8(12):e84361. doi: 10.1371/journal.pone.0084361 (PMC3877289; doi:10.1371/journal.pone.0084361)
Supplement: Table S1 — Minimum Inhibitory Concentration to polymyxin B and tobramycin in the isolates used in this study. (DOCX) [file pone.0084361.s001.docx]

| **Isolate** | **MIC for polymyxin B (µg/mL)^a^** | **MIC for tobramycin (µg/mL)^b^** |
| --- | --- | --- |
| 1 | 0.5 | 4 |
| 2 | 0.5 | ND^c^ |
| 3 | 0.5 | ND^c^ |
| 5 | 0.5 | ND^c^ |
| 6 | 0.5 | 2 |
| 7 | 0.5 | 4 |
| 8 | 0.5 | 2 |
| 11 | 2 | 0.5 |
| 14 | 2 | 2 |
| 19 | 0.5 | ND^c^ |
| 20 | 0.5 | 4 |
| 25 | 0.5 | 4 |
| 28 | 0.5 | 1 |
| 29 | 2 | ND^c^ |
| 31 | 1 | 2 |
| 32 | 2 | 4 |
| 33 | 2 | 0.5 |
| 34 | 1 | 0.5 |
| 35 | 2 | 2 |
| 36 | 0.5 | 2 |
| 37 | 0.5 | 4 |
| 39 | 0.5 | ND^c^ |
| 40 | 0.5 | 0.5 |
| 45 | 0.5 | 0.5 |
| 47 | 2 | 1 |
| 48 | 1 | ND^c^ |
| 49 | 2 | 0.5 |
| 52 | 2 | 1 |
| 55 | 2 | 0.5 |
| 56 | 2 | 4 |
| 57 | 0.5 | 2 |
| 61 | 0.5 | 2 |
| 62 | 0.5 | 2 |
| 65 | 0.5 | 2 |
| 66 | 0.5 | 2 |
| 68 | 0.5 | 1 |
| 75 | 0.5 | 4 |

^a^ MIC range to be considered susceptible: ≤ 2 µg/mL

^b^ MIC range to be considered susceptible: ≤ 4 µg/mL

^c^ ND: Not Determined. Strains were previously classified as resistant in the disk diffusion test.
